# Anti-Telomerase CD4^+^ Th1 Immunity and Monocytic-Myeloid-Derived-Suppressor Cells Are Associated with Long-Term Efficacy Achieved by Docetaxel, Cisplatin, and 5-Fluorouracil (DCF) in Advanced Anal Squamous Cell Carcinoma: Translational Study of Epitopes-HPV01 and 02 Trials

**DOI:** 10.3390/ijms21186838

**Published:** 2020-09-17

**Authors:** Laurie Spehner, Stefano Kim, Angélique Vienot, Eric François, Bruno Buecher, Olivier Adotevi, Dewi Vernerey, Syrine Abdeljaoued, Aurélia Meurisse, Christophe Borg

**Affiliations:** 1 INSERM, EFS BFC, UMR1098, RIGHT, University of Bourgogne Franche-Comté, Interactions Greffon-Hôte-Tumeur/Ingénierie Cellulaire et Génique, F-25000 Besançon, France; LAURIE.SPEHNER@efs.sante.fr (L.S.); chkim@chu-besancon.fr (S.K.); angelique.vienot@inserm.fr (A.V.); olivier.adotevi@univ-fcomte.fr (O.A.); abdeljaoued.syrine@hotmail.fr (S.A.); 2 Department of Medical Oncology, University Hospital of Besançon, F-25000 Besançon, France; 3 Department of Medical Oncology, North Franche-Comté Hospital, F-25200 Montbéliard, France; 4 Clinical Investigational Center, CIC-1431, F-25000 Besançon, France; dvernerey@chu-besancon.fr (D.V.); ahusse@chu-besancon.fr (A.M.); 5 Oncology Multidisciplinary Group (GERCOR), F-75011 Paris, France; 6 French Federation of Digestive Cancerology (FFCD), F-21000 Dijon, France; 7 Department of Medical Oncology, Antoine-Lacassagne Center, F-06100 Nice, France; eric.francois@nice.unicancer.fr; 8 Department of Medical Oncology, Curie Institute, F-75005 Paris, France; bruno.buecher@curie.fr; 9 Methodology and Quality of Life in Oncology Unit, University Hospital of Besançon, F-25000 Besançon, France

**Keywords:** advanced anal squamous cell carcinoma, DCF chemotherapy, M-MDSC, hTERT antigens, Biomarkers

## Abstract

Docetaxel, cisplatin and 5-fluorouracil (DCF) chemotherapy regimen is highly effective in advanced anal squamous cell carcinoma (SCCA), as demonstrated by the Epitopes-HPV02 study results. Here, we analyzed the impact of DCF regimen and the prognostic value of adaptive immune responses and immunosuppressive cells in SCCA patients included in two prospective studies (Epitopes-HPV01 and HPV02). The presence of T-cell responses against Human papillomavirus (HPV)16-E6/E7 and anti-telomerase (hTERT)-antigens was measured by IFNᵧ-ELISpot. Here, we showed that HPV-adaptive immune responses are increased in SCCA patients. SCCA patients also displayed enhanced circulating TH1 T-cells restricted by hTERT. Exposition to DCF increased hTERT immunity but not HPV or common viruses immune responses. Notably, the correlation of hTERT immune responses with SCCA patients’ clinical outcomes highlights that hTERT is a relevant antigen in this HPV-related disease. The influence of peripheral immunosuppressive cells was investigated by flow cytometry. While both regulatory T-cells and monocytic-myeloid-derived suppressive cells (M-MDSC) accumulated in the peripheral blood of SCCA patients, only high levels of M-MDSC were negatively correlated with hTERT adaptive immune responses and predicted poor prognosis. Altogether, our results reveal that hTERT is a relevant antigen in HPV-driven SCCA disease and that M-MDSC levels influence TH1-adaptive immune responses and patients’ survival.

## 1. Introduction

Anal squamous cell carcinoma (SCCA) is a rare disease accounting for 2.7% of gastrointestinal malignancies, but the incidence rate of this cancer is increasing each year worldwide [1]. SCCA is associated with E6 and E7 oncoproteins encoded by Human Papillomavirus (HPV) observed in 90% of cases [2,3]. HPV16 is the most prevalent genotype reported in this disease. Treatment of advanced SCCA remains a challenging issue since complete remission is a rare event, observed in less than 5% of cases and treatment of metastatic diseases are performed in a palliative setting.

We have recently shown that the addition of docetaxel to cisplatin plus 5-fluorouracil (DCF) might be an attractive strategy for advanced SCCA patients, generating high levels of long-term complete remissions (Epitopes-HPV02, NCT02402842) [4]. Indeed, 25% of the patients treated with DCF remained free of progression in an updated analysis of this study [5]. These promising results prompted us to explore the biological mechanisms underlying DCF efficacy.

Docetaxel, a microtubule-stabilizing agent, exerts cytotoxic functions by blocking dividing cells in G2/M phase, leading to apoptosis. Docetaxel-based chemotherapy has also been involved in the modulation of anti-tumour immune responses. Indeed, docetaxel was described to induce calreticulin, a damage-associated molecular patterns (DAMPs) related to the immunogenic cell death (ICD) [6]. Sobering observations indicated that another possible influence of docetaxel might be an inhibition of immunosuppressive cells, sustaining the potential restoration of an effective anti-tumour immunity [7].

To address this hypothesis and considering the particular clinical benefits observed in SCCA patients included in Epitopes-HPV01 and HPV02 trials, we decided to investigate the impact of DCF chemotherapy on peripheral immunological parameters in these patients. Here we presented the correlations of DCF efficacy outcomes with specific immune responses and immunosuppressive cell levels, and then determined the prongnostic value of myeloid suppressive cells in SCCA patients treated by DCF chemotherapy.

## 2. Results

### 2.1. Magnitude of Anti-Telomerase (hTERT)-Specific T Helper 1 (TH1) Response was Increased in SCCA Patients Following DCF Chemotherapy

**Figure 1:**
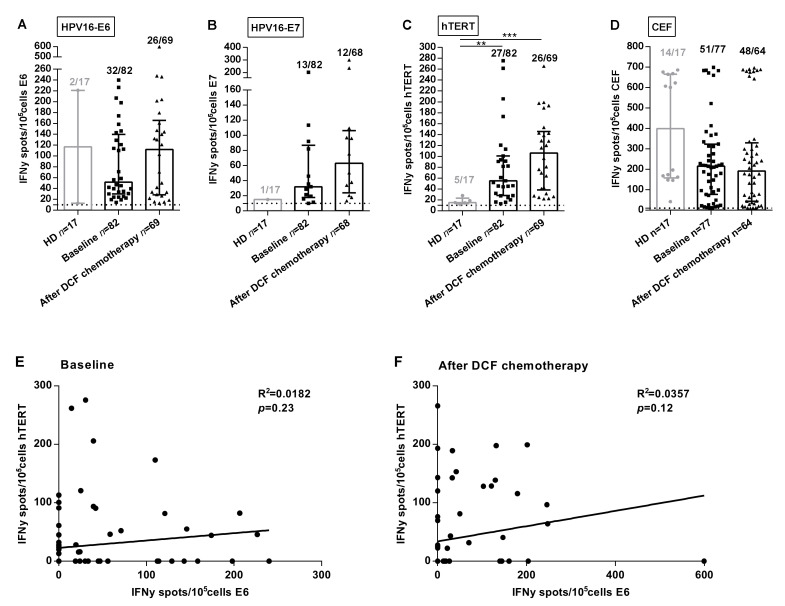
Frequencies and intensities of antigen-specific T-cell responses in SCCA patients. PBMC from 17 healthy donors and SCCA patients before (*n* = 82) and after (*n* = 69) DCF chemotherapy were analyzed for antigen-specific T-cell responses by IFNᵧ ELISpot assay. A-C Intensity of positive HPV16-E6 (**A**), HPV16-E7 (**B**) and hTERT (**C**) specific T-cell responses in healthy donors and SCCA patients before and after DCF treatment. (**D**) Intensity of positive antiviral T-cell responses. Healthy donors population is represented by light gray points and SCCA patients by black points. Mann Whitney *U* test, where ** *p* < 0.01, *** *p* < 0.001. Median with interquartile range was indicated on graphs. Only the positive intensities of specific immune responses were indicated. (**E**,**F**) Correlation between HPV16-E6- and hTERT-specific immune responses before (**E**) and after DCF chemotherapy (**F**) in SCCA patients.

HPV-associated cancers are a convenient model to investigate the clinical implications of antigen-specific T-cell responses since E6 and E7 immunogenic peptides are well characterized and processed in most HPV-positive patients. Moreover, E6AP ubiquitin ligase binds to hTERT promoter activating hTERT gene transcription [8]. Thus, we speculated that hTERT might be an antigen in HPV-related cancers. In order to analyze SCCA-related specific immune responses, ELISpot assays were used to measure Interferon-gamma (IFNᵧ) produced by CD8 and CD4 T-cells recognizing E6 and E7 peptides. We also took advantage of telomerase promiscuous peptides characterized in our team and presented in most HLA-DR contexts to specifically monitor antigen-specific TH1 cells [9]. Experiments were conducted using PBMC collected in two prospective clinical trials investigating the role of DCF in advanced or metastatic first line SCCA patients. The median intensity and frequency of HPV- and hTERT-specific immune responses were increased in SCCA patients. At baseline, percentages of SCCA patients with reactive T lymphocytes were 39.0%, 15.9% and 32.9% compared with 11.8%, 5.9% and 29.4% in healthy volunteers for E6, E7 and hTERT, respectively (*p* = 0.031, *p* = 0.28, *p* = 0.77; Figure 1A–C). Moreover, the median intensity of hTERT-specific TH1 responses at baseline was stastistically enhanced in SCCA patients compared to healthy volunteers (55.0 SFC/10^5^ cells (IQR: 28.0–100.5) vs. 15.0 SFC/10^5^ cells (IQR: 13.0–23.25), *p* = 0.0015, Figure 1C). This first set of experiments suggest that SCCA patients develop spontaneous anti-HPV adaptive immunity detectable in their peripheral blood. Moreover, our results suggest that telomerase is a candidate antigen in HPV-driven carcinoma. Of note, E6 and hTERT immune responses were not correlated when measured before (Figure 1E) or after treatment (Figure 1F).

Interestingly, the median intensity of hTERT-specific TH1 responses was enhanced following DCF (106.0 SFC/10^5^ cells (IQR: 38.3–145.5) vs. 55.0 SFC/10^5^ cells (IQR: 28.0–100.5), *p* = 0.16), as for E6 (112 SFC/10^5^ cells (IQR: 28.4–165.5) vs. 51.5 SFC/10^5^ cells (IQR: 30.1–139.5), *p* = 0.50), but to a lesser extent for E7 (63 SFC/10^5^ cells (IQR: 23.9–63.0) vs. 32 SFC/10^5^ cells (IQR: 17.8–87.0), *p* = 0.22; Figure 1A–C). However, the antiviral recall T-cell responses were detected with high intensities in the majority of SCCA patients before (215.5 SFC/10^5^ cells (IQR: 77.5–323.5), 66.2%) and after DCF chemotherapy (192.5 SFC/10^5^ cells (IQR: 44.4–330.5), 75%) (Figure 1D). Also, DCF did not modulate common viruses-restricted T cell immunity, suggesting a possible interaction between this docetaxel-based chemotherapy and the modulation of the measured antigen T-cell responses. The modulations of specific immune responses (increase, decrease or absence) observed in patients whose samples were available before and after treatment is depicted in Supplementary Figure S1A–D.

### 2.2. Peripheral Antigen-Specific T Cells Are Correlated with SCCA Patients’ Survival

Next, we analyzed if peripheral T lymphocytes recognizing HPV-related antigens were correlated with SCCA patients’ outcomes.

Characteristics of SCCA patients exhibiting peripheral antigen-specific T cells are shown in Table 1. The presence of HPV-specific immune responses and hTERT-specific immune repsonses was not influenced by the main clinical characteristics of the patients included (Table 1).

**Figure 2:**
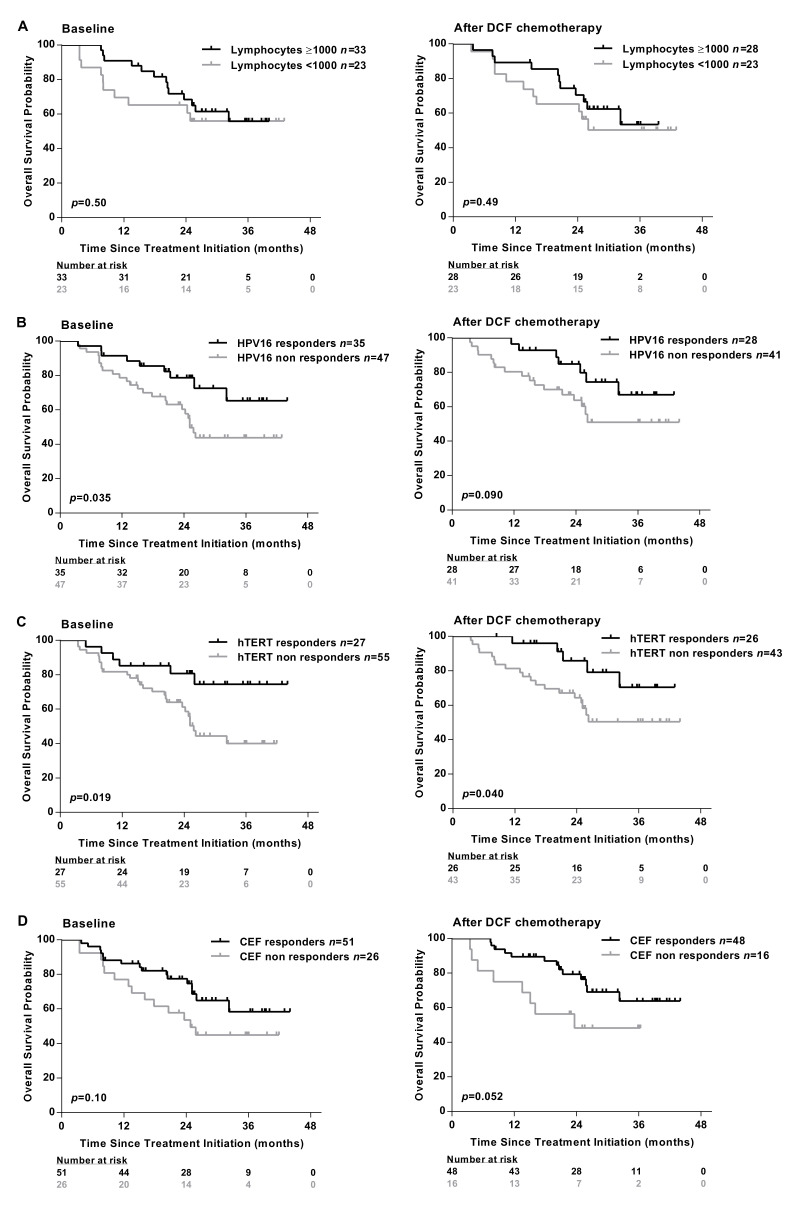
Presence of antigen-specific T-cell responses are associated with the clinical outcomes of SCCA patients. (**A**) The absolute lymphocyte count was analyzed in peripheral blood of SCCA patients before (*n* = 56) and after (*n* = 51) DCF chemotherapy. Kaplan-Meier curve in SCCA patients before and after DCF chemotherapy according to low absolute lymphocyte count. PBMC from SCCA patients before (*n* = 82) and after (*n* = 69) DCF chemotherapy were analyzed for antigen-specific T-cell responses by IFNᵧ ELISpot assay. (**B**,**C**) Kaplan-Meier OS curve in SCCA patients according to HPV (E6 and/or E7) (**B**) or hTERT (**C**) specific T-cell responses before and after DCF chemotherapy. Log-rank test, where *p* < 0.05. (**D**) Kaplan-Meier OS curve in SCCA patients according to antiviral-specific T-cell responses before and after DCF chemotherapy.

Absolute lymphocyte count measured at baseline did not associate with specific immune responses (Table 1) and did not predict SCCA patients’ survival before and after treatment (Figure 2A).

Conversely, an improved overall survival (OS) was observed in patients exhibiting HPV, hTERT-restricted immune responses measured before or after DCF chemotherapy (Figure 2B,C). As expected, the presence of immune responses recognizing common viruses (EBV, CMV, influenza) was not correlated with survival (Figure 2D).

To better elucidate the underlying determinants of antigen-specific immune responses in these patients, an immune profiling of peripheral CD4 and CD8 lymphocytes was performed by flow cytometry (Supplementary Figure S2A). CD4 and CD8 T-cell activation was estimated using OX40 and 4-1BB, respectively. OX40 and 4-1BB expression did not differ according to the detection of antigen-specific immune responses in the peripheral blood (Table 1). PD1 was expressed on 8.0% and 5.0% of CD4 and CD8 T-cells both in patients with or without HPV/hTert immune responses (Table 1). CD226 and TIGIT expression also displayed similar levels of expression in these patients (Table 1).

Exposition to DCF had no impact on co-activatory/inhibitory receptor expression on CD4 and CD8 T-cells. Therefore, we hypothesized that DCF might act through the depletion of immunosuppressive cells.

### 2.3. Influence of Peripheral Monocytic Myeloid-Derived Suppressive Cells (M-MDSC) and Regulatory CD4^+^ T-Cells (Treg) on the Clinical Outcomes of SCCA Patients

**Figure 3:**
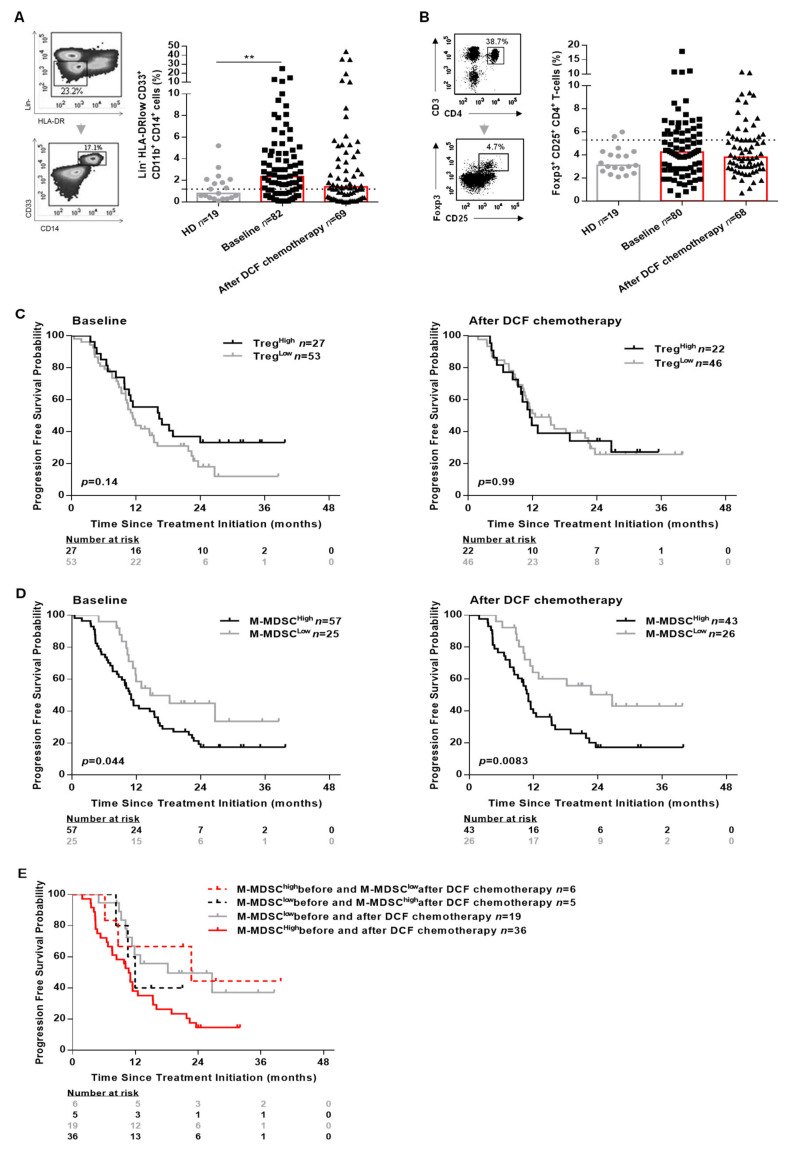
M-MDSC levels and not Treg levels are associated with the clinical outcomes in SCCA patients. PBMC from 19 healthy donors and SCCA patients before (*n* = 82) and after (*n* = 69) DCF chemotherapy were analyzed for Treg and M-MDSC population by flow cytometry. (**A**) Frequencies (%) of M-MDSC. (**B**) Frequencies (%) of CD25^+^Foxp3^+^ expressed on CD4^+^ T-cells. (**C**) Kaplan-Meier survival curve in SCCA patients before and after DCF chemotherapy according to Treg levels. (**D**) Kaplan-Meier survival curve in SCCA patients before and after DCF chemotherapy according to M-MDSC levels. Log-rank test, where ** *p* < 0.01. (**E**) Kaplan-Meier curve in distribution of M-MDSC levels in SCCA patients treated by DCF chemotherapy.

The precise role of immune suppressive cells was not thoroughly investigated in SCCA. We therefore assessed the percentages of circulating M-MDSC and Treg using flow cytometry (Figure 3A,B, Supplementary Figure S2B,C). M-MDSC were defined as HLA-DR^low^Lin^-^CD11b^+^CD33^+^CD14^+^ according to previous reports [10], Treg cells were defined as CD3^+^CD4^+^CD25^+^Foxp3^+^.

Both M-MDSC (2.4% vs. 0.8%, *p* = 0.0060) and Treg (4.2% vs. 3.1%, *p* = 0.07) levels were increased in SCCA patients compared to healthy donors. Interestingly, only M-MDSC levels reached statistical significance (Figure 3A). The influence of peripheral Treg and M-MDSC on SCCA patients’ survival was investigated using a threshold (light gray broken line in Figure 3A,B) determined with statistical methods (Supplementary Figure S3). Using the maximizing of log-rank test, we selected 5.3% as threshold for Treg and 1.2% for M-MDSC (Supplementary Figure S3). SCCA patients’ survival was not correlated to peripheral Treg levels (Figure 3C and Supplementary Figure S4A). By contrast, high M-MDSC levels were significantly associated with a shorter OS of SCCA patients whether measured before (median OS: not achieved vs. 25.2 months, *p* = 0.0017) or after DCF chemotherapy (median OS: not achieved vs. 26.0 months, *p* = 0.0054; Supplementary Figure S4B). Additionally, high M-MDSC levels were significantly predictive of a shorter PFS at baseline (median PFS: 14.6 vs. 11.0 months, *p* = 0.044) and after DCF chemotherapy (median PFS: 26.7 vs. 11.0 months, *p* = 0.0083) (Figure 3D). DCF decreased M-MDSC levels in 35 out of 66 patients (2.5% vs. 1.2%, *p =* 0.0012) but did not modulate Treg levels. Interestingly, no change in monocyte levels was observed compared to healthy donors before and after treatment. In addition, monocyte levels were not correlated with OS nor PFS (Supplementary Figure S5), sustaining the specific impact of M-MDSC on SCCA pateints’ survival.

Of note, SCCA patients exhibiting high M-MDSC levels at baseline and low M-MDSC levels after DCF chemotherapy had a better survival compared to SCCA patients with high M-MDSC levels before and after DCF chemotherapy (median PFS: 22.7 vs. 10.1 months, *p* = 0.04; Figure 3E). Based on these findings, we can state that high M-MDSC levels but not Treg levels are predictive of poor prognosis in SCCA.

### 2.4. DCF Chemotherapy Alleviated M-MDSC Suppression of hTERT TH1 Immunity

**Figure 4:**
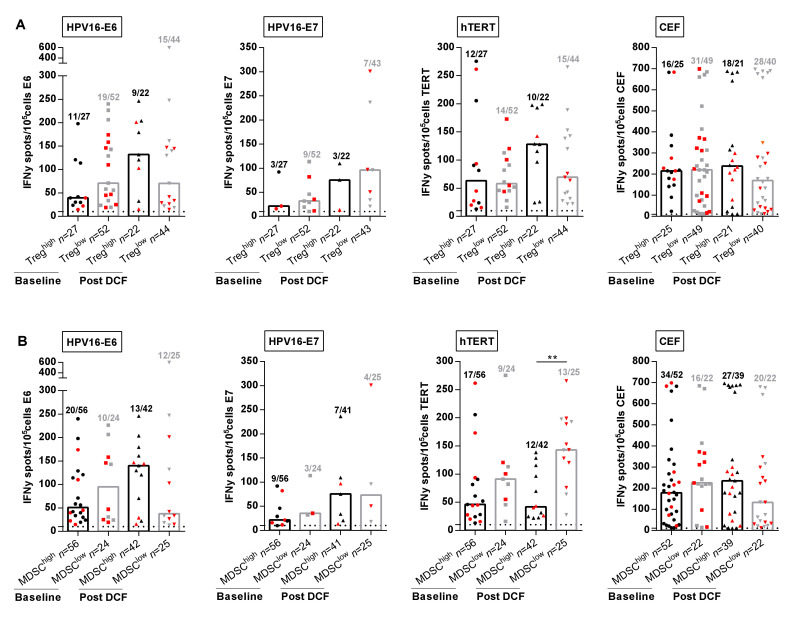
M-MDSC and not Treg levels are correlated with antigen-specific immune responses. (**A**,**B**) Intensities of positive HPV16-E6-, HPV16-E7-, hTERT- and antiviral-specific T-cell responses in SCCA patients before and after DCF chemotherapy according to Treg levels (**A**) or M-MDSC levels (**B**). Mann Whitney *U* test, where ** *p* < 0.01. Red plots represent patients without progression.

In the next set of experiments, we decided to investigate the correlation between immune suppressive cell levels and specific adaptive immune responses. Previous studies reported that in vitro depletion of Treg results in increased HPV-specific T-cell responses in cervical neoplasia [11]. However, frequencies and intensities of HPV-specific T-cell responses were not impaired by high levels of Treg in SCCA patients before or following DCF chemotherapy (Figure 4A).

We next evaluated the influence of M-MDSC levels on peripheral immune responses. The percentages of patients with peripheral lymphocytes responding to E6, hTERT and common viral antigens had decreased in the presence of high M-MDSC levels (Figure 4B). Strikingly, DCF selectively enhanced the frequency (28.6% vs. 52.0%, *p* = 0.055) and the intensity (143.0 SFC/10^5^ cells (IQR: 98.0–195.3) vs. 41.8 SFC/10^5^ cells (IQR: 24.9–110.8), *p* = 0.0012) of hTERT TH1 immune responses only when M-MDSC levels were below 1.2% (Figure 4B). In addition, 8 out of 13 patients with hTERT TH1^high^ and M-MDSC^low^ immune profile measured after DCF were free of progression compared to three of 12 patients with hTERT TH1^low^ and M-MDSC^high^. No similar correlation could be established for E6, E7 or common viral immune responses, suggesting that hTERT was an antigen of particular interest in SCCA disease.

## 3. Discussion

Docetaxel-based chemotherapy was recently established as a therapeutic option leading to long term survival in metastatic or advanced SCCA [4]. Immune checkpoint inhibitors are other candidate therapies that are gaining momentum in improving SCCA patients’ clinical outcomes [12,13]. Thus, combining DCF to anti-Programmed cell death-1 (PD-1) or anti-Programmed death-ligand 1 (PD-L1) might be an attractive approach in this disease. Consequently, we conducted a study to describe the impact of DCF on SCCA patients’ immunity. Here, we have provided evidence that DCF chemotherapy did not hamper the level of lymphocyte counts, Treg or antigen-specific immune responses. Moreover, the high intensities and frequencies of antiviral recall of T-cell responses reported in Epitopes-HPV01 and HPV02 studies were comparable to those observed in NSCLC or colorectal cancer patients [14,15] and were not modulated by DCF chemotherapy. In contrast, our results show that DCF could decrease M-MDSC levels.

The prognostic value of specific immune responses in the peripheral blood of cancer patients have been reported in several malignancies [15,16,17]. Masterson et al. also demonstrated that the presence of E7-specific immune responses in the peripheral blood of HPV^+^ head and neck squamous cell carcinoma (HNSCC) patients was associated with better OS [18]. The presence of HPV16-E6/E7- and hTERT-specific T-cells was investigated for the first time in patients with SCCA. An HPV-specific immunity was detectable in the peripheral mononuclear cells of SCCA patients (Figure 1). The presence of HPV-specific T-cells is prognostic as revealed by a better OS (Figure 2). However, the impact of HPV adaptive immunity on DCF efficacy is dismal since the modulation of E6- and E7-specific T-lymphocytes by DCF is not significant, even in patients displaying low levels of M-MDSC (Figure 4). Conversely, hTERT-driven TH1 immune responses were increased following DCF, particularly in patients where M-MDSC levels were low (Figure 4). Here, we demonstrated that the presence of hTERT-specific immune responses in peripheral blood of SCCA patients is a prognostic factor.

Several studies have demonstrated that E6 and MYC interaction leads to the transactivation of hTERT promoter [19]. In line with the direct activation of hTERT expression by E6 oncoprotein, we could observe an increased frequency of hTERT-restricted CD4 TH1 lymphocytes in the PBMC of SCCA patients indicating that hTERT was a relevant antigen in HPV diseases.

Previous results also suggested that HPV-driven antigens were not the only source of tumour-associated antigens in HPV-related cancers, but also Stevanović et al. indicated that mutated neoantigens or cancer germline antigens are immunodominant T-cell epitopes in HPV-related cancers [20]. Here, we demonstrated that hTERT-derived peptides are also a source of epitopes leading to T-cell reactivity and correlated with SCCA clinical outcomes. We identified hTERT as a novel tumor-associated antigen in HPV^+^ SCCA.

We recently observed that the addition of anti-PD-1 or anti-PD-L1 blocking was able to restore the function of hTERT-specific CD4^+^ Th1 response in lung cancer patients treated by Nivolumab [15]. The role of PD-1 and PD-L1 in immune evasion of HPV-associated tumors was well described in HNSCC [21,22]. These results might pave the way for innovative therapeutic vaccination strategies for HPV^+^ SCCA in particular and HPV driven cancers in general. We have recently initiated a phase I/II study combining hTERT Th1 inducing vaccine and anti-PD-L1 (NCT03946358).

The presence of a potent immunosuppressive cells such as Treg and M-MDSC might be another determinant of SCCA disease. Increased levels of Treg in peripheral blood of cancer patients have been reported in several malignancies but not in SCCA patients [23]. Nevertheless, it was reported that Foxp3 expression was associated with progression of cervical cancer [24]. Indeed, the up-regulation of Foxp3 was correlated with the p16^INK4a^ expression, an important protein for the HPV integration and was associated with the tumor growth. If previous studies have revealed that high Treg levels are associated with poor survival in patients with gastric, esophageal [23] or ovarian cancers [25], recent studies have reported that high Treg levels have no impact on OS in HPV^+^ HNSCC tumors [26]. Interestingly, our results sustained these findings; although an increase of Treg levels was observed in peripheral blood of patients with SCCA, it was neither correlated with adaptive immunity nor clinical outcomes.

Circulating MDSC has been widely reported as a poor prognosis biomarker in several solid tumors. In addition, there is a growing body of evidence suggesting that MDSC plays a key role in dampening the anti-tumor immune responses. To the best of our knowledge, this is the first study to investigate the clinical implications of M-MDSC in SCCA after DCF chemotherapy. We found that M-MDSC levels were significantly increased in SCCA patients’ peripheral blood compared to healthy donors. Moreover, high M-MDSC levels but neither Treg nor monocytes were markedly associated with poor OS as well as PFS. Remarkably, DCF was able to decrease M-MDSC levels below the threshold in 6 out of the 42 patients classified as M-MDSC^high^ at baseline and induced a better survival in these patients (Figure 3E). Nevertheless, functional studies should be conducted in order to decipher the underlying mechanisms behind this observation. The promotion of immunogenic cell death of HPV^+^ cancers by docetaxel-based chemotherapy is a possible hypothesis sustaining the raise of such adaptive immunity. Indeed, docetaxel induces calreticulin exposure, one of three DAMPs associated to the ICD [27]. Not withstanding, the presence of 5-fluorouracil in the DCF regimen might also contribute to the modulation of MDSC levels [28,29].

The ability of DCF to deplete MDSC in some patients while generating hTERT restricted TH1 immune response established the rational to combine this chemotherapy with PD-1 neutralization by an inhibitor of PD-L1 in HPV-driven cancers. Such enticing approach is currently under investigation (A Study of mDCF in Combination or Not With Atezolizumab in Advanced Squamous Cell Anal Carcinoma (SCARCE), NCT03519295) [30].

This study emphasized the key role of hTERT-specific CD4^+^ Th1 responses and M-MDSC levels as prognostic factors to better stratify SCCA patients’ risk of death.

## 4. Methods

### 4.1. Study Design and Participants

Epitopes-HPV01 was a prospective cohort study performed by the regional cancer network of Franche-Comté, including one university hospital, five community hospitals, and one private center. Epitopes-HPV02 trial was a single-arm phase II study supported by the Oncology Multidisciplinary Group (GERCOR) and French Federation of Digestive Cancerology (FFCD) collaborative oncological groups and performed in 25 French academic hospitals [4]. In both studies, SCCA patients with metastatic diseases or unresectable local recurrences were enrolled in the same criteria. These trials were registered with ClinicalTrials.gov (https://clinicaltrials.gov/) numbers NCT01845779 and NCT02402842. Epitopes-HPV01 was reviewed and approved by the independent Est-II French Committee for Protection of Persons on 9 July 2012, and by the French Health Products Safety Agency on 6 July 2012. Epitopes-HPV02 was approved by the independent Est-II French Committee for Protection of Persons on 6 June 2014, and by the French Health Products Safety Agency on 15 July 2014. 

HPV genotype was characterized in HPV02 trial by two different methodologies. ctDNA of HPV16 derived E7 was investigated in 57 patients and identified E7 DNA in 52 of them (91.1%). HPV genotype was also characterized in tumor biopsies using Inno-LiPA assay. Fifty-seven out of 61 patients were HPV16 positive (93.4%) and 2 patients were HPV33 positive. We failed to observe HPV DNA in two patients. Regarding the low number of patients with HPV negative tumor and the risk of false negative related to molecular technique used, it was decided to analyze immune responses in all patients. Blood samples of SCCA patients were analyzed at baseline (21 patients included in Epitopes-HPV01 and 63 patients included in Epitopes-HPV02) and three months after DCF chemotherapy (21 patients included in Epitopes-HPV01 and 49 patients included in Epitopes-HPV02). A flowchart describing the selection process for patients included in the clinical and translational studies was included in Supplementary Figure S6. For healthy volunteers (*n* = 19), blood cells were collected in an anonymous manner (EFS, Bourgogne-Franche-Comté, France) as apheresis kit preparation after the signature of informed consent and according to EFS guidelines. The major exclusion criteria for blood collection in healthy donors were minors (<18 years), age over 65 years, dehydration, fatigue, low levels of hemoglobin (>120 g/L for women and 130 g/L for men), flu symptoms, HIV, HTLV or hepatitis B/C positive status, autoimmune diseases, surgical procedures in the last 4 months and vaccine less than 4 weeks old.

### 4.2. Synthetic Peptides

Peptides covering E6 and E7 oncoproteins encoded by HPV16 were purchased from Miltenyi Biotec (Bergisch Gladbach, Germany). PepTivator peptide pools consisting of 15-mer sequences with 11 amino acid overlap, covering the complete sequences of the HPV16-E6 and E7 protein. Therefore, immune responses measured by PepTivator-derived HPV peptides represent both CD4 and CD8 T-cells. We used a mix of eight promiscuous HLA-class II-binding 15-mer peptides derived from Telomerase to specifically monitor Th1 CD4 T-cells responses described by our team (TERT_44–58_, TERT_541–555_, TERT_573–587_, TERT_578–592_, TERT_613–627_, TERT_911–925_, TERT_921–935_, TERT_1055–1069_) (patent US9669080B2) [9,15,31]. Synthetic peptides (>80% purity) were purchased from JPT peptide technologies and were reconstituted in 5% of Dimethyl sulfoxide (DMSO, WAK-Chemie Medical GmbH, Steinbach, Germany) and Phosphate Buffer Saline (PBS, Gibco, Illkirch, France) at a concentration of 4 mg/mL.

### 4.3. Assessment of Antigen Specific T-Cell Responses in Healthy Donors and SCCA Patients

Peripheral blood mononuclear cells (PBMC) from SCCA patients and healthy donors were isolated by density centrifugation on Ficoll gradient (Eurobio, Courtaboeuf, France). PBMC of SCCA patients were cryopreserved at a cell density of 8–15 × 10^6^ cells per vial in CryoStor (CS10 and CS5) cell preservation media (Sigma-Aldrich, St. Louis, MO, USA) and were conserved at −196 °C for flow cytometry and ELISpot analysis.

Frozen PBMC (viability > 70%) were seeded at 4 × 10^6^ cells per well in a 24-well plate in complete medium (RPMI supplemented with 10% human serum, 10,000 UI/mL penicillin and 10,000 μg/mL streptomycin, Gibco, Illkirch, France) and were exposed to PepTivator HPV16-E6 and E7 (1 μg of each peptide/mL) and the pool of peptides derived from Telomerase (5 μg/mL). A pool of the 23 peptides containing epitopes from CEF (Cytomegalovirus, Epstein-Barr and Influenza virus) (2 μg of each peptide/mL) (Cellular Technology Limited, Shaker heights, OH, USA) was used to evaluate antiviral responses. Recombinant interleukin (IL)-7 (5 ng/mL) and IL-2 (20 UI/mL, Peprotech, Neuilly-sur-seine, France) were added at days 1 and 4, respectively. At day 7, after a short-term in vitro stimulation, antigen-specific T-cell responses were monitored by IFNᵧ Enzyme-Linked ImmunoSpot (ELISpot) assay (Diaclone, Besançon, France).

### 4.4. ELISpot Assay

IFNᵧ ELISpot protocol was adapted from Godet et al. [9]. Briefly, T-cells (10^5^ cells per well) were cultured in anti-human IFNᵧ monoclonal antibody precoated ELISpot plate with PepTivator HPV16 E6 and E7 (1 μg of each peptide/mL), the pool of peptides derived from Telomerase (5 μg/mL) and the pool of peptides derived from CEF (2 μg of each peptide/mL) in X-Vivo 15 medium (Lonza, Bâle, Switzerland) for 17–18 h at 37 °C. Cells cultured with medium alone or Phorbol-12-myristate-13-acetate/Ionomycin (250 ng/mL; 10 μg/mL, Sigma-Aldrich, St. Louis, MO, USA) were used as negative and positive controls, respectively. All experiments were conducted in duplicates and each result presented is the mean of the duplicates. The IFNᵧ’s spots were revealed following the manufacturer’s instructions (Diaclone, Besançon, France). Estimation of specific T-cell number was expressed as spot-forming cells (SFC)/10^5^cells and calculated after subtracting negative control values (background). Spot-forming cells were counted using the C.T.L Immunospot system (Cellular technology limited, Shaker heights, OH, USA) and assessed with Immunospot 5.0 analyser software (Bonn, Germany). Responses were considered as positive when IFNᵧ spot number was ≥10 and ratio 2-fold above background.

### 4.5. Flow Cytometry

For surface staining, PBMC were washed and stained for 30 min at 4 °C in PBS/30% human serum with the following Fixable viability Dye (FvD)-eFluor 780 (eBioscience, Villebon-sur-Yvette, France) and antibodies. Immune checkpoints expressions were investigated performing surface staining with CD3-Pacific-blue (clone UCHT1; BD biosciences, Franklin Lakes, NJ, USA), 4-1BB-APC (clone 4B4-1; BD biosciences), CD8-PercpCy5.5 (clone SK1; BD biosciences), PD-1-BV510 (clone EH12.1; BD biosciences), CD4-FITC (clone B-A1; Diaclone), TIGIT-APC (clone MBSA43; eBioscience), CD226-PercpCy5.5 (clone 10E5; Biolegend, San Diego, CA, USA), TIM3-PeCy7 (Clone F38-2E2; Biolegend) and CTLA4-PE (clone BNI3; BD biosciences). M-MDSC and monocytes were characterized by surface staining using negative lineage (Lin-) Pacific blue (CD3, CD56 and CD19) (clone OKT3, HCD56 and SJ25C1; Biolegend), CD14-PE (clone M5E2; BD biosciences), CD33-APC (clone WM53; BD biosciences), CD11b-PeCy7 (clone ICRF44; BD biosciences) and anti-HLA-DR-FITC (clone B-F1; Diaclone). For Treg analysis, T-cells were first stained with surface antibodies CD3-Pacific blue (clone UCHT1; BD biosciences), CD4-FITC (clone B-A1; Diaclone) and CD25-PeCy7 (clone MA-251; BD biosciences). Intracellular staining was performed following the manufacturer’s instructions (BD biosciences). T-cells were fixed and permeabilized with Human Foxp3 buffer set and then stained with Foxp3-PE (clone 259D/C7; BD biosciences). Samples were directly acquired on a Facs Canto II (BD biosciences) and analyzed with DIVA software Franklin Lakes, NJ, USA).

### 4.6. Statistical Analysis

Statistical analyses were performed using GraphPad Prims 6 software (San Diego, CA, USA). The level of significance was set at *p* < 0.05 for all tests (* *p* ≤ 0.05, ** *p* ≤ 0.01, *** *p* ≤ 0.001 and **** *p* ≤ 0.0001). Variables were expressed as a median and interquartile range (IQR) and tested with the Mann-Whitney *U* test. Variables were expressed as frequencies and tested with the Fisher’s exact test.

Overall survival (OS) was calculated from the date of the first administration of chemotherapy to the date of death from any cause. Survival data were censored at the last follow-up. Progression-free survival (PFS) was calculated from the date of the first administration of chemotherapy to the date of progression or death from any cause, or the date of the last follow-up, at which point data were censored. OS and PFS were estimated using the Kaplan-Meier method and compared using the log-rank test.

To give a reasonable spread of risk in Treg and M-MDSC populations, we distinguished two prognostic groups according to the maximizing of the log-rank test, which was determined following the Hothorn and Lausen method (R package “maxstat”) [32]. These cut-offs were validated by the restricted cubic splines method with graphical evaluation. Analyses to determine thresholds were performed using R software version 3.5.3 (R Development Core Team; http://www.r-project.org).

## Figures and Tables

**Table 1:** Patients’ characteristics in overall population and according to HPV or hTERT immune responses in the peripheral blood at baseline.

	Overall Population *n* = 82	HPV Non Responders *n =* 47	HPV Responders *n* = 35	*p*-Value *	hTERT Non Responders *n =* 55	hTERT Responders *n* = 27	*p*-Value ^**^
Genre							
Female	63 (76.8%)	37 (78.7%)	26 (74.3%)	0.64	42 (76.4%)	21 (77.8%)	0.88
Male	19 (23.2%)	10 (21.3%)	9 (25.7%)		13 (23.6%)	6 (22.2%)	
Age							
Median [IQR]	60.0 [38.6–84.0]	59.4 [38.6–78.4]	61.7 [41.1–84.0]	0.27	60.1 [38.6–84.0]	59.5 [44.2–78.4]	0.81
ECOG							
0	53 (64.6%)	31 (66.0%)	22 (62.9%)	0.89	36 (65.4%)	17 (63.0%)	0.87
1	28 (34.2%)	15 (31.9%)	13 (37.1%)		18 (32.7%)	10 (37.0%)	
2	1 (1.2%)	1 (2.1%)	0 (0.0%)		1 (1.8%)	0 (0.0%)	
HIV positif							
Positive	78 (95.1%)	44 (93.6%)	34 (97.1%)	0.63	52 (94.5%)	26 (96.3%)	1.0
Negative	4 (4.9%)	3 (6.4%)	1 (2.9%)		3 (5.6%)	1 (3.7%)	
Stage							
Locally advanced	17 (20.7%)	10 (21.3%)	7 (20.0%)	0.71	12 (21.8%)	5 (18.5%)	0.34
Synchronous metastases	22 (26.8%)	11 (23.4%)	11 (31.4%)		12 (21.8%)	10 (37.0%)	
Metachronous metastases	43 (52.4%)	26 (55.3%)	17 (48.6%)		31 (56.4%)	12 (44.4%)	
Number of sites involved							
Median [IQR]	2.0 [1.0–8.0]	2.0 [1.0–8.0]	2.0 [1.0–5.0]	0.84	2.0 [1.0–8.0]	2.0 [1.0–5.0]	0.65
Previous Chemoradiotherapy	(*n* = 48)	(*n* = 29)	(*n* = 19)		(*n* = 36)	(*n* = 11)	
MMC + Cape/5FU	36 (78.3%)	22 (75.9%)	14 (82.3%)	0.34	26 (74.3%)	10 (90.9%)	0.38
CDDP + 5FU	6 (13.0%)	5 (17.2%)	1 (5.9%)		6 (17.1%)	0 (0.0%)	
Capecitabine	1 (2.2%)	1 (3.4%)	0 (0.0%)		1 (2.9%)	0 (0.0%)	
Missing	2	0	2		1	1	
RT without CT							
No	73 (93.6%)	41 (93.2%)	32 (94.1%)	1.0	51 (96.2%)	22 (88.0%)	0.32
Yes	5 (6.4%)	3 (6.8%)	2 (5.9%)		2 (3.8%)	3 (12.0%)	
Missing	4	3	1		2	2	
Absolute lymphocyte count	(*n* = 59)	(*n* = 35)	(*n* = 21)		(*n* = 37)	(*n* = 19)	
Median [IQR]	1086.0 [860.0–1700.0]	1159.0 [875.0–1927.0]	1019.0 [850.0–1411.0]	0.28	1159.0 [820.0–1869.0]	1019.0 [875.0–1600.0]	0.56
Immune checkpoints expression on CD4 T-cells							
OX40	14.5 [9.6–24.1]	13.7 [7.4–20.8]	17.6 [9.5–26.7]	0.27	13.2 [7.9–23.3]	17.6 [9.5–26.6]	0.35
CD226^+^ TIGIT^−^	34.1 [24.1–44.3]	32.8 [23.9–42.8]	35.0 [24.1–44.8]	0.79	34.4 [24.2–42.4]	31.5 [23.6–45.1]	0.81
CD226^+^ TIGIT^+^	12.2 [7.9–17.0]	11.5 [7.8–15.8]	14.5 [8.8–18.9]	0.12	12.1 [7.9–13.2]	14.2 [7.4–20.6]	0.36
CD226^−^ TIGIT^+^	8.1 [5.6–10.6]	7.8 [5.4–9.8]	8.3 [6.4–11.3]	0.27	8.1 [5.6–10.8]	7.7 [6.4–10.1]	0.93
PD-1	8.6 [6.0–13.0]	8.9 [5.8–14.2]	8.9 [6.7–12.3]	0.79	9.4 [6.1–13.5]	8.1 [5.0–11.8]	0.37
Missing	0	1	1		1	1	
Immune checkpoints expression on CD8 T-cells							
4-1BB	0.1 [0.0–0.3]	0.1 [0.0–0.4]	0.1 [0.0–0.2]	0.41	0.1 [0.0–0.4]	0.1 [0.0–0.2]	0.18
CD226^+^ TIGIT^−^	36.6 [22.0–49.1]	39.3 [23.0–47.3]	35.9 [21.0–52.4]	0.82	39.7 [23.3–54.2]	27.4 [21.0–42.4]	0.05
CD226^+^ TIGIT^+^	17.6 [10.4–27.5]	17.8 [10.3–26.3]	16.1 [10.3–24.6]	0.71	14.7 [8.7–28.6]	17.2 [14.0–29.4]	0.24
CD226^−^ TIGIT^+^	15.9 [9.5–28.8]	14.7 [9.3–28.0]	19.0 [10.7–28.8]	0.28	16.3 [9.6–23.0]	19.3 [12.9–34.4]	0.06
PD-1	5.0 [3.3–8.7]	5.3 [3.8–9.4]	4.8 [2.6–7.9]	0.12	5.0 [3.6–8.8]	5.1 [2.9–8.9]	0.83
Missing	0	1	1	1	1	1	

* Medians and proportions were compared between HPV-responders and HPV non-responders using Wilcoxon–Mann–Whitney and χ^2^ tests (or Fisher’s exact test, if appropriate) respectively; ** Medians and proportions were compared between hTERT-responders and hTERT non-responders using Wilcoxon–Mann–Whitney and χ^2^ tests (or Fisher’s exact test, if appropriate) respectively. 5FU, 5-Fluorouracil; Cape, Capecitabine; CDDP, Cisplatin; ECOG-PS, Eastern Cooperative Oncology Group performance status; HIV, Human Immunodeficiency Virus; MMC, Mitomycin.

## Supplementary Materials

The following are available online at https://www.mdpi.com/1422-0067/21/18/6838/s1.
