# Supplementary material for: Anti-Telomerase CD4+ Th1 Immunity and Monocytic-Myeloid-Derived-Suppressor Cells Are Associated with Long-Term Efficacy Achieved by Docetaxel, Cisplatin, and 5-Fluorouracil (DCF) in Advanced Anal Squamous Cell Carcinoma: Translational Study of Epitopes-HPV01 and 02 Trials
Source: Int J Mol Sci. 2020 Sep 17;21(18):6838. doi: 10.3390/ijms21186838 (PMC7554943; doi:10.3390/ijms21186838)
Supplement: Supplementary file 1 [file ijms-21-06838-s001.pdf]

Supplementary Materials

Anti-Telomerase CD4+ Th1 Immunity and Monocytic-Myeloid-Derived-Suppressor Cells are Associated with Long-Term Efficacy Achieved by Docetaxel, Cisplatin, and 5-Fluorouracil (DCF) in Advanced Anal Squamous Cell Carcinoma: Translational Study of Epitopes-HPV01 and 02 Trials

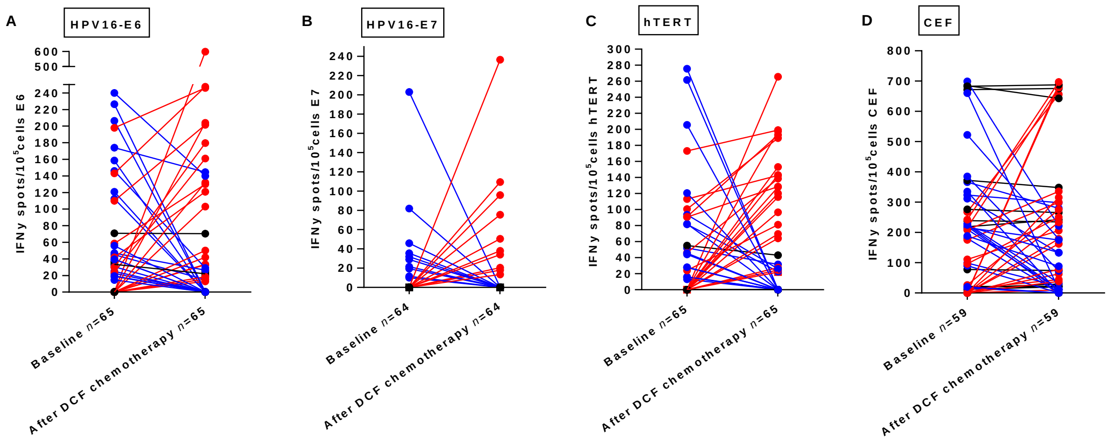

Figure S1. The overall effect of DCF treatment on the antigen-specific T-cell responses in SCCA patients. PBMC from SCCA patients whose samples were available before and after DCF chemotherapy (*n* = 65 for E6 and hTERT, *n* = 64 for E7 and *n* = 59 for CEF) were analyzed for antigen-specific T-cell responses by IFN$_\gamma$ ELISpot assay. (**A**–**D**) The linking individual plot was used to follow the intensity of HPV16-E6 (**A**), HPV16-E7 (**B**), hTERT (**C**) and antiviral T-cell responses (**D**) in SCCA patients before and after treatment. Increase specific-immune responses are represented by red gray points, decrease specific-immune responses by blue points and absence of immune responses by black points.

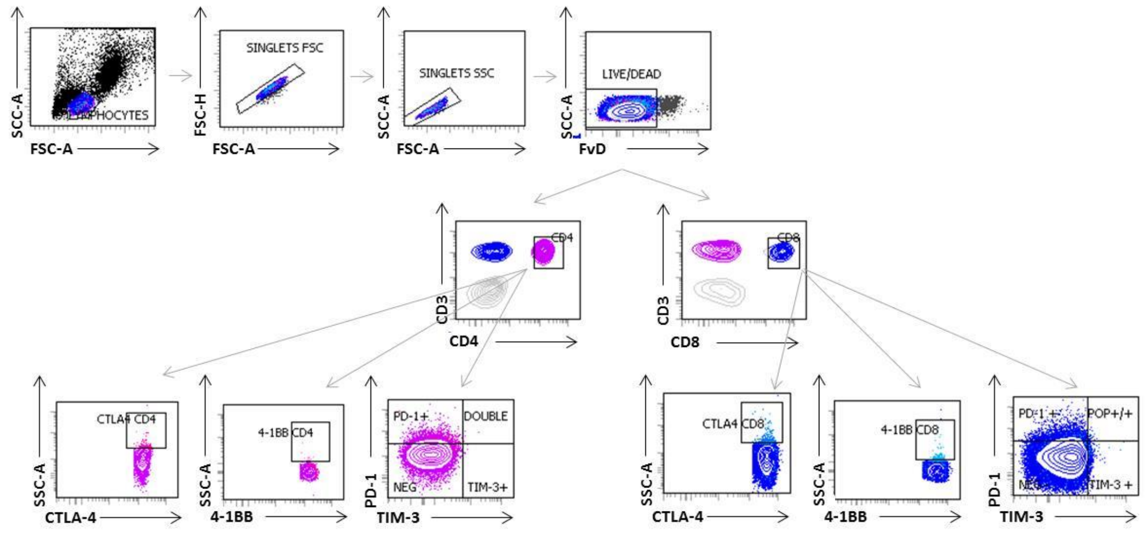

A

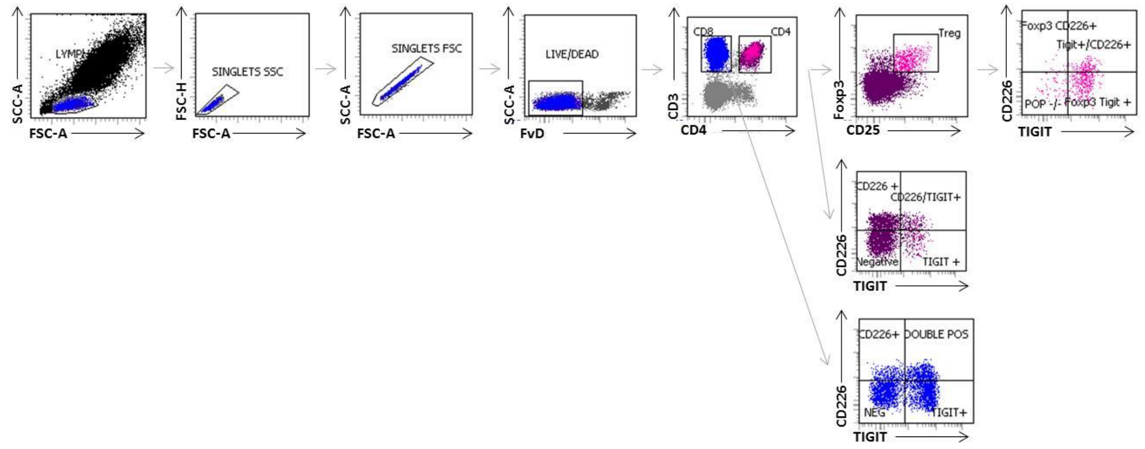

B

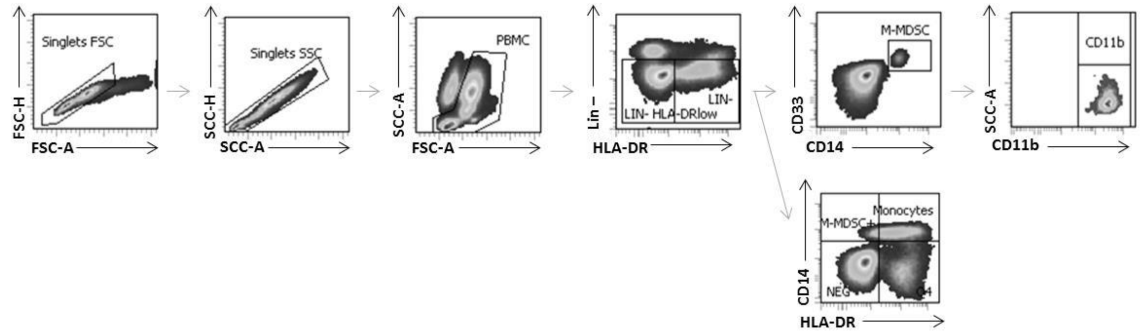

C

Figure S2. Gating strategy for flow cytometry analyses. The figure shows the gating strategy to analyze immune checkpoints (**A**), Treg (**B**) or M-MDSC (**C**) populations. After exclusion of doublets and death cells, CD4 and CD8 T-cell populations were sectioned. The expression of CTLA-4 (or OX40), 4-1BB, PD-1 and TIM-3 were analyzed on CD4 and CD8 T-cells (**A**). Frequencies of Treg cells were observed in CD4 T-cell population. Expression of CD226 and TIGIT were analyzed on CD4, Treg and CD8 T-cells (**B**). M-MDSC and monocyte populations were analyzed after exclusion of lineage (CD3, CD56, CD19) and among PBMC (**C**).

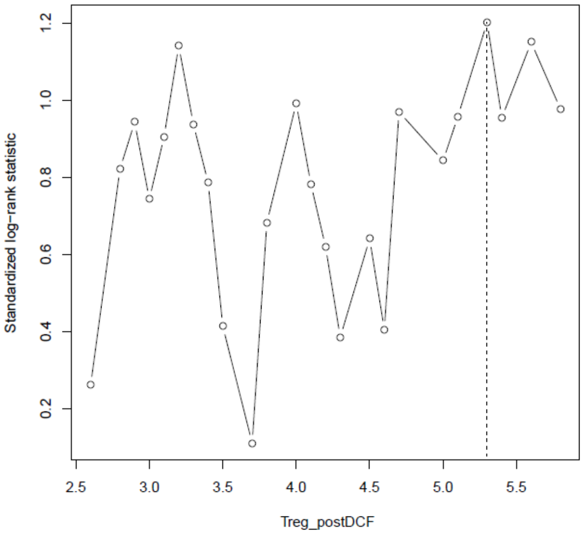

A

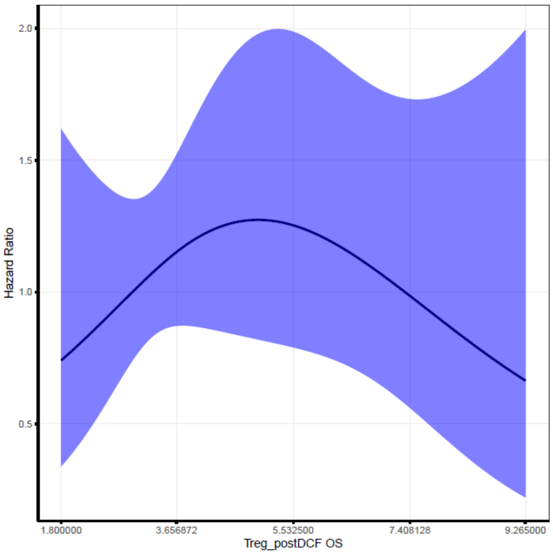

B

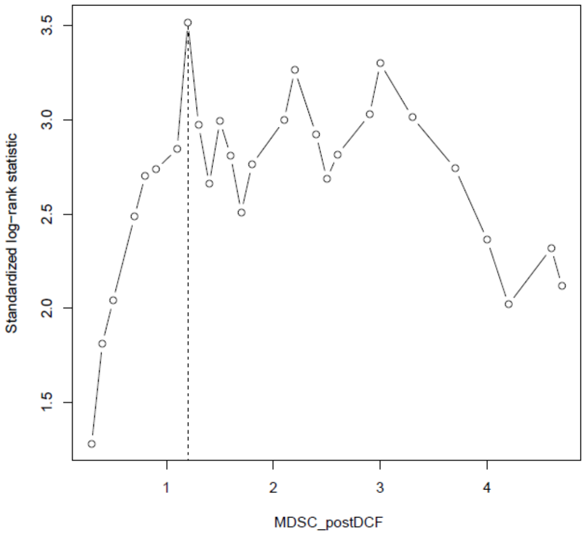

C

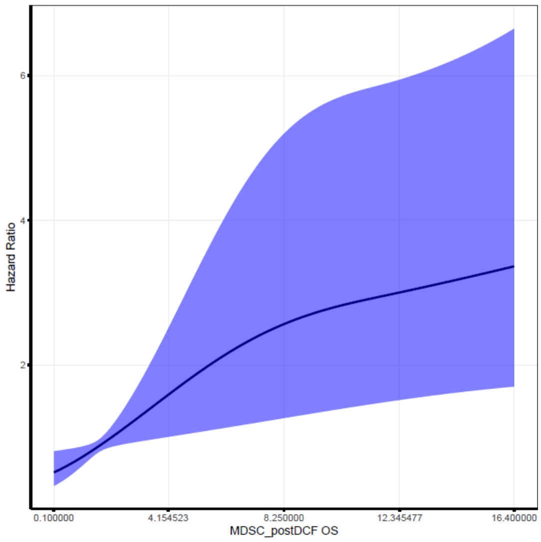

D

Figure S3. Determining thresholds for Treg and M-MDSC populations. The thresholds of Treg (**A**) and M-MDSC (**C**) populations after DCF treatment were chosen with maximizing of the log-rank test (maxstat cutoff). The relationship between overall survival and Treg (**B**) and M-MDSC (**D**) distributions was also investigated using the restricted cubic splines method with graphical evaluation. The selected thresholds were used to analyze the association of Treg and M-MDSC high subpopulations with immune responses and survival of SSCA patients.

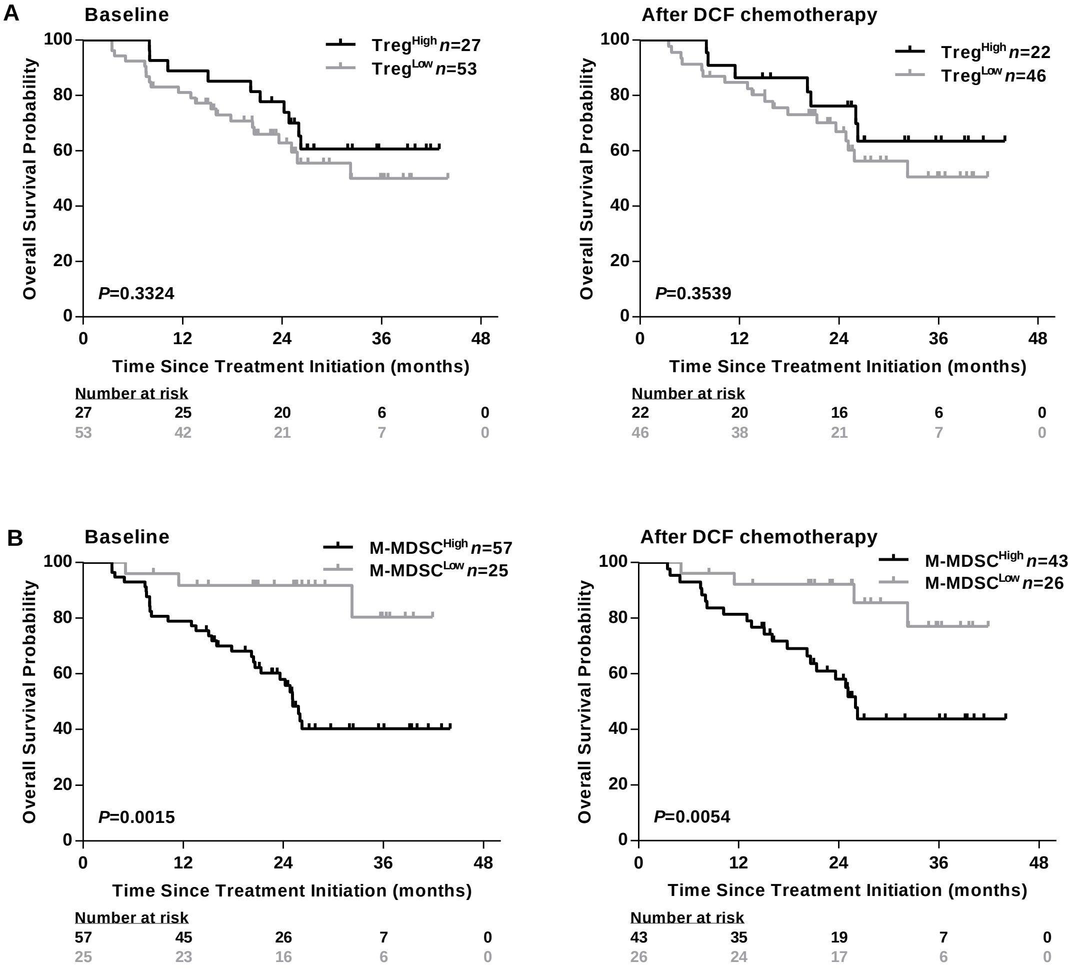

Figure S4. M-MDSC levels and not Treg levels are associated with the clinical outcomes of SCCA patients. PBMC from 19 healthy donors and SCCA patients before (n = 82) and after (n = 69) DCF chemotherapy were analyzed for Treg and M-MDSC populations by flow cytometry. A Kaplan-Meier OS curve in SCCA patients before and after DCF chemotherapy according to Treg levels. B Kaplan-Meier OS curve in SCCA patients before and after DCF chemotherapy according to M-MDSC levels. Log-rank test, where ** $p < 0.01$.

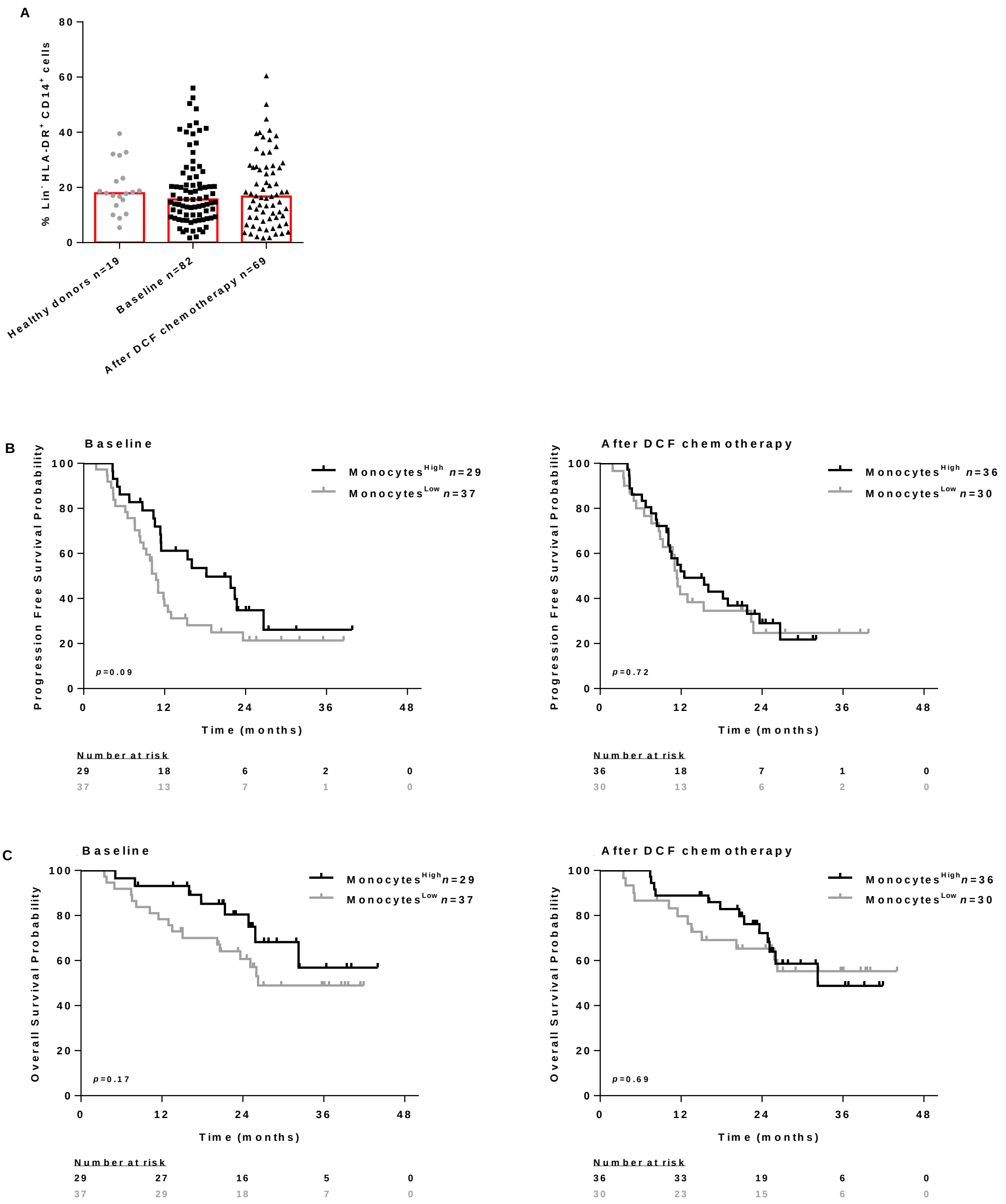

Figure S5. Monocyte levels are not associated with the clinical outcomes of SCCA patients. PBMC from 19 healthy donors and SCCA patients before (n = 82) and after (n = 69) DCF chemotherapy were analyzed for monocytes populations by flow cytometry. (**A**) Frequencies (%) of monocytes. We selected a median at the threshold (16.1%) to separate into 2 groups our patients. (**B**) Kaplan-Meier survival curve in SCCA patients before and after DCF chemotherapy. (**C**) Kaplan-Meier OS curve in SCCA patients before and after DCF chemotherapy according to monocytes levels.

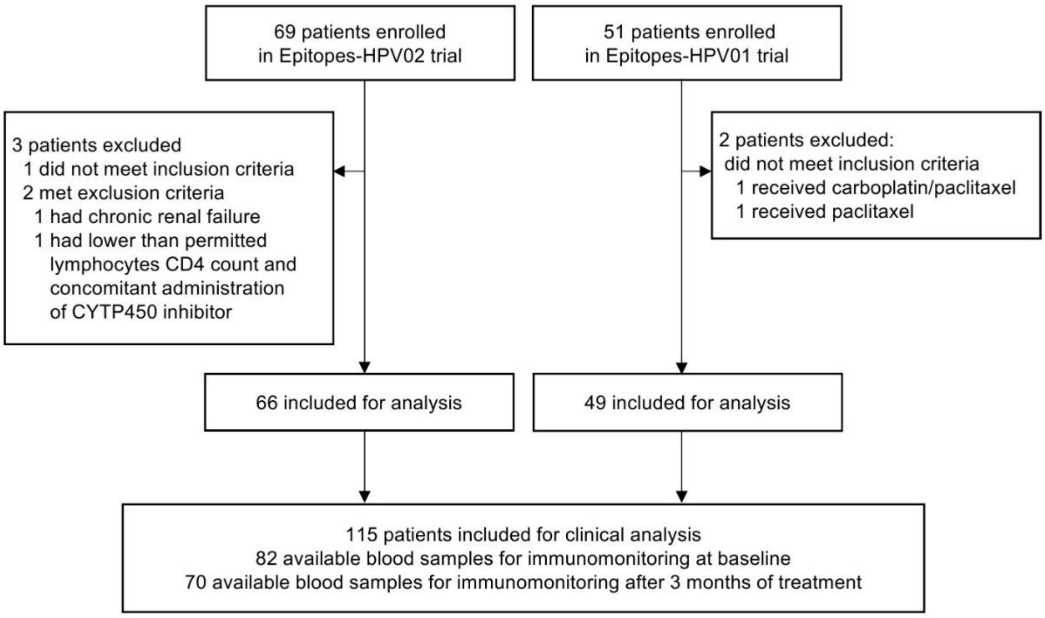

Figure S6. Flowchart. DCF = docetaxel, cisplatin and 5-fluorouracil.
